# Delaying task failure in high-intensity exercise: a Pi-afferent-effort framework for targeted sports nutrition

**DOI:** 10.1080/15502783.2026.2711031

**Published:** 2026-07-30

**Authors:** Jeffrey R. Stout, Terry J. Housh, Haley C. Bergstrom

**Affiliations:** a School of Kinesiology and Rehabilitation Sciences, University of Central Florida, Orlando, FL, USA; b Department of Nutrition and Health Sciences, University of Nebraska-Lincoln, Lincoln, NE, USA

**Keywords:** Inorganic phosphate, phosphocreatine, peripheral fatigue, group, III/IV afferents, perceived effort, high-intensity, exercise

## Abstract

**Background:**

Task failure during high-intensity exercise may reflect the convergence of intramuscular, neural, perceptual, and behavioral boundaries rather than the consequence of a single fatigue mechanism.

**Methods:**

This hypothesis-generating narrative review proposes the Pi-afferent-effort model of task failure during high-intensity exercise and applies the model to targeted performance nutrition.

**Results:**

Rapid ATP turnover increases inorganic phosphate (Pi), while phosphocreatine breakdown through the creatine kinase reaction buffers ATP availability. As phosphate-linked disturbance progresses, Pi may impair crossbridge function, Ca^2+^ sensitivity, and excitation-contraction coupling and thereby reduce force capacity. In parallel, interstitial acid-base disturbance and metabolite- and mechanosensitive group III/IV afferent feedback support ventilatory and circulatory regulation, but may also constrain motor output and voluntary activation. Perceived effort and muscle pain are treated as distinct constructs: effort is closely related to central motor command, whereas pain and discomfort reflect nociceptive-affective processing influenced by afferent feedback and context. Nutritional strategies are interpreted according to the model boundary they are most likely to affect. Nitrate may reduce ATP cost and phosphate-linked perturbation in selected tasks; creatine may support PCr availability and between-bout recovery; beta-alanine and sodium bicarbonate may alter intracellular and extracellular acid-base stress; caffeine may affect arousal, motor output, effort appraisal, pain, and tolerance; and carbohydrate is most relevant during prolonged or repeated high-intensity work.

**Conclusions:**

Future studies should combine metabolic, neuromuscular, perceptual, and performance outcomes to determine whether supplements reduce work-matched strain, increase tolerance of terminal strain, or both.

## 1. Introduction

Task failure during high-intensity exercise is not usually attributable to a single event. A cyclist who cannot sustain a severe-intensity power output, a runner who fails late in a middle-distance race, a combat athlete who cannot maintain repeated flurries, or a team-sport player who loses repeated sprint capacity is not simply out of ATP, full of lactate, insufficiently motivated, or limited by one supplement-responsive pathway. The endpoint is determined by the interaction among the contractile state of the active muscle, neural drive, cardiorespiratory regulation, sensory feedback, perceived effort, pain, pacing, and task-specific behavioral tolerance.

Sports nutrition interventions are often discussed as products with broad performance claims. Nitrate may improve exercise economy or tolerance in selected contexts. Creatine can enhance repeated high-intensity performance. Beta-alanine increases skeletal-muscle carnosine after chronic loading. Sodium bicarbonate can increase blood bicarbonate and pH and thereby support extracellular buffering and H^+^ efflux. Caffeine improves several performance outcomes, and carbohydrate supports endurance and repeated high-intensity work. These statements are useful; however, they do not fully explain why an intervention is effective in one high-intensity phenotype and not another. The central premise of the present review is that high-intensity performance nutrition should be interpreted according to the boundary it modifies: phosphate-linked contractile disturbance, acid-base and afferent feedback, central motor output, perceived effort, pain, substrate availability, or behavioral tolerance.

One difficulty in this area is the inconsistent use of fatigue terminology. Enoka and Duchateau [1] proposed that fatigue should be understood through the interaction between performance fatigability and perceived fatigability, rather than as a single phenomenon with a fixed anatomical locus. Hunter [2] emphasized that performance fatigability is task-specific and determined by the demands imposed on the neuromuscular system. Gandevia [3] demonstrated that human muscle fatigue does not reside only within the muscle, because spinal and supraspinal processes can reduce voluntary drive. Neurochemical central-fatigue hypotheses, including serotonin-dopamine interactions during prolonged exercise, are relevant to sports nutrition but should not be used as synonyms for voluntary activation, motivation, or perceived effort [4]. Cellular mechanisms within the muscle fiber remain central to high-intensity exercise, particularly when ATP turnover is high, and phosphagen and glycolytic flux produce substantial metabolite disturbance [5].

The critical power framework provides a useful example of this integration. Below critical power, muscle metabolites such as phosphocreatine (PCr), Pi, and pH can stabilize after an initial perturbation. Above critical power, PCr and Pi generally do not reach a steady state, and pH disturbance often progresses. The magnitude and time course of H^+^ accumulation, however, depend on exercise mode, fiber recruitment, and glycolytic contribution [6,7]. During intermittent high-intensity exercise, recovery intervals allow partial restoration of PCr and related phosphate-linked metabolites, which increases the amount of work that can be performed above critical power [8]. These observations are directly relevant to nutrition, because many ergogenic aids do not increase maximal force or motivation in isolation. Instead, they may alter the rate at which fatigue-related boundaries are reached.

The present review focuses on continuous severe-intensity exercise, repeated sprint exercise, high-intensity interval training, and sport contexts that combine repeated bursts with incomplete recovery. It does not attempt to explain all forms of fatigue and does not imply that the same mechanism dominates isolated strength exercise, low-intensity endurance exercise, or recovery from chronic training stress. Therefore, the purpose of this review was threefold: to synthesize evidence linking intramuscular Pi, acid-base stress, group III/IV afferent feedback, central motor inhibition, perceived effort, pain, and task failure; to evaluate common nutritional strategies as mechanistic interventions within this pathway; and to propose testable predictions for future work using integrated physiological and perceptual endpoints [9–11].

## 2. Narrative review approach

This paper provides a hypothesis-based narrative review rather than the detailed approach of a systematic review, meta-analysis, or ISSN position stand. Searches were updated in May 2026 and performed using PubMed, Web of Science, and by cross-referencing key reviews, mechanistic papers, relevant position stands, and systematic reviews and meta-analyses on supplementation and high-intensity exercise. Search terms included: inorganic phosphate (Pi), phosphocreatine (PCr), 31P MRS, Pi speciation, skeletal muscle fatigue, cross-bridge function, excitation-contraction coupling (ECC), group III/IV muscle afferents, voluntary activation (VA), perception of effort (PoE), rating of perceived exertion (RPE), muscle pain, sensory tolerance, beta-alanine (βA), sodium bicarbonate (NaHCO3), caffeine (Caf), creatine (Cr), dietary nitrate (NO3−), carbohydrate (CHO), repeated sprint exercise (RSE), severe-intensity exercise, high-intensity interval training (HIIT), combat sport, and team sport. The paper preferentially emphasized studies using 31P MRS, evoked contractile measures, VA assessment methods, methods that can manipulate or attenuate afferent input to the spinal cord and brain, or controlled supplementation study designs. Position stands, systematic reviews, and meta-analyses were prioritized for supplement-specific recommendations.

In contrast, original mechanistic work was preferentially selected if the topic was Pi, pH, cross-bridge dynamics, Ca^2+^ handling, afferent feedback, or VA. This review prioritized supplements that have a plausible mechanism of action in line with the Pi-afferent-effort pathway and are widely used in sports nutrition practice: NO_3_^−^, Cr, βA, NaHCO_3_, Caf, and CHO. This review excluded supplements where the proposed ergogenic mechanism did not involve failure at high intensity; studies with primarily clinical evidence and not focused on sports performance; or when available evidence did not support a clear mechanistic link with the framework. The review did not perform risk-of-bias assessments or grading of evidence, nor did it pool data using meta-analysis; therefore, any conclusions drawn should be regarded as hypothesis-generating rather than exhaustive. To minimize overinterpretation of the proposed model and highlight tractable research avenues, this review demarcated areas supported by direct evidence, those inferred through plausibility, and those not yet addressed.

## 3. Conceptual scope and terminology

Because several constructs are often conflated, the terminology used in the present review should be explicit. Fatigue is used as a broad symptom or state that emerges from interactions between objective changes in performance capacity and subjective sensations that regulate task continuation [1]. Performance fatigability refers to an acute decline in an objective measure such as force, power, work rate, sprint output, maximal voluntary contraction, or time to task failure [1,2]. Peripheral fatigue refers to reduced force or power capacity caused by processes at or distal to the neuromuscular junction. During high-intensity exercise, these processes may involve crossbridge function, Ca^2+^ release, myofibrillar Ca^2+^ sensitivity, sarcolemmal excitability, and sarcoplasmic reticulum function [5]. Sarcolemmal excitability can also be influenced by changes in K^+^, Na^+^, and Cl^−^ gradients and channel conductance; therefore, peripheral fatigue should not be attributed only to Pi or H^+^ [12].

Central fatigue refers to reduced voluntary neural drive to the muscle due to spinal or supraspinal processes. It may be assessed with twitch interpolation, transcranial magnetic stimulation, cervicomedullary stimulation, or related estimates of voluntary activation [3]. Voluntary activation is not equivalent to motivation, sleepiness, or the broad feeling of tiredness. Thus, a motivated participant may still show reduced voluntary activation if neural feedback or central inhibitory processes constrain motoneuronal output.

Group III and IV muscle afferents are thinly myelinated and unmyelinated sensory neurons that respond to contraction-related mechanical deformation and interstitial chemical stimuli, including combinations of H^+^/pH disturbance, lactate, ATP, K^+^, bradykinin, prostaglandins, and related metabolites [9,10]. They should not be described as a simple pain pathway or as direct sensors of intramyocellular Pi. Some populations function as non-nociceptive ergoreceptors during normal exercise, whereas stronger or noxious stimuli can recruit metabo-nociceptive pathways that contribute to pain, discomfort, and inhibitory motor effects. Whole-body cycling studies with afferent attenuation link this feedback to constrained motoneuronal output and intramuscular metabolic perturbation [11].

Perceived effort is the conscious sensation of how hard, heavy, or strenuous a voluntary action feels. When narrowly defined, it is strongly associated with central motor command and corollary discharge rather than a direct readout of peripheral metabolite concentration [13–15]. Broad Borg RPE or CR10 ratings may capture a mixture of effort, pain, breathlessness, discomfort, and fatigue sensations, depending on scale instructions [16,17]. Muscle pain is distinct from perceived effort. It is a nociceptive and affective sensation influenced by metabolite-sensitive and mechanosensitive afferent feedback, central processing, expectation, prior experience, and context [3,9,10,15,16]. Exercise tolerance is the amount of work or duration that can be sustained under a given task constraint. Task failure may reflect mechanical inability to produce the required force or power, but it can also reflect disengagement when effort, pain, risk, or perceived cost exceeds tolerance.

## 4. The Pi-afferent-effort model of high-intensity exercise task failure

The Pi-afferent-effort model proposes that high-intensity exercise task failure occurs when peripheral contractile capacity, afferent feedback, central motor drive, perceived effort, muscle pain, and behavioral tolerance converge on a task-specific endpoint. In this framework, intramuscular Pi is treated primarily as a contractile and phosphate-linked metabolic variable, not as the direct sensory signal to group III/IV afferents. The afferent signal is attributed to the interstitial and mechanical conditions that develop with high ATP turnover, PCr depletion, glycolytic flux, and acid-base disturbance.

The model includes three primary boundaries. The first is intramuscular. ATP hydrolysis generates Pi, whereas the creatine kinase system buffers ATP as PCr falls. The coupled decline in PCr and increase in free Pi mark phosphagen disturbance during severe-intensity exercise. As Pi accumulates, it can impair myofibrillar force production, reduce Ca^2+^ sensitivity, and contribute to impaired sarcoplasmic reticulum Ca^2+^ release and excitation-contraction coupling, particularly when other fatigue-related changes in Ca^2+^ handling and activation are present [5–7,18].

The second boundary is neural. Mechanical deformation or tension, interstitial H^+^/pH disturbance, lactate, ATP, K^+^, bradykinin, prostaglandins, and related vascular or interstitial chemical changes create a chemical and mechanical milieu that stimulates group III/IV afferents [9,10]. This feedback helps regulate ventilation and circulation, but it can also constrain motoneuronal output and central motor drive. When lower-limb group III/IV feedback was attenuated during whole-body cycling, motoneuronal output increased, while exercise-induced Pi, H^+^, ADP, lactate, PCr depletion, and quadriceps twitch fatigue were greater [11]. Thus, the response is context-dependent regulation rather than a process that is simply protective or limiting.

The third boundary is perceptual and behavioral. Rising central motor command contributes to perceived effort. Metabolite-sensitive afferent feedback contributes to pain and other aversive sensations. Task continuation depends on whether the required motor command, pain, and perceived cost remain tolerable relative to goals, expectations, risk, and task constraints [13–17].

The model should not be interpreted to mean that Pi is the only cause of fatigue or that supplements should eliminate Pi. Pi is required for ATP resynthesis and is an essential component of energy metabolism. The practical target is Pi kinetic control. This may include slowing Pi accumulation, delaying the crossing of a functional Pi threshold, reducing Pi exposure above that threshold, improving PCr/Pi recovery between bouts, or altering the consequences of Pi accumulation through improved acid-base regulation, central drive, or tolerance.

### 4.1. Evidence status and limits of inference

Because the Pi-afferent-effort model integrates evidence from reduced preparations, intact muscle, whole-body exercise, afferent attenuation studies, perceptual research, and supplementation trials, the strength of inference differs across model links. Direct support is strongest for Pi effects on contractile function, PCr/Pi/pH kinetics during severe exercise, and the role of group III/IV feedback in regulating motor output during whole-body exercise. Inferences are needed when nutritional strategies are proposed to reduce afferent escalation by altering upstream metabolic or acid-base stress. Hypotheses are indicated when supplement combinations, phenotype-specific threshold shifts, or direct afferent effects have not been tested.

## 5. Mechanistic basis of the model

### 5.1. Intramuscular Pi and phosphate-linked peripheral fatigue

During high-intensity exercise, ATP turnover increases rapidly. The creatine kinase reaction buffers ATP by transferring a phosphate group from PCr to ADP, thereby producing ATP and creatine. PCr breakdown does not directly release free Pi. Rather, ATP hydrolysis yields Pi while the creatine kinase reaction maintains ATP availability. With continued severe-intensity work, this coupling is expressed as decreasing PCr, increasing Pi, decreasing pH, and an intracellular environment that becomes progressively less favorable for force production [5–8].

Pi appears to affect muscle function at several levels. In skinned fibers and myofibrils, elevated Pi lowers force and alters crossbridge transitions, consistent with a shift of crossbridge states away from force-generating configurations and toward detachment. The inhibitory phosphate species may also be pH-dependent. Diprotonated phosphate (H_2_PO_4_−) has been linked to force depression in skinned fast skeletal muscle fibers, but this interpretation should be qualified because it is derived largely from reduced preparations at nonphysiological temperatures and is not uniformly established across fiber types or physiological temperatures. Pi can reduce myofibrillar Ca^2+^ sensitivity and, in later fatigue, may contribute to impaired excitation-contraction coupling and reduced sarcoplasmic reticulum Ca^2+^ release. The Ca^2+^-Pi precipitation mechanism remains plausible and indirectly supported, but it should not be presented as definitively proven in exercising human muscle [18–21].

Support for the importance of Pi is provided by isolated fiber, intact muscle, human 31P-MRS, and modeling studies. Allen and Westerblad [19] argued that Pi has a central role in fatigue, particularly through effects on Ca^2+^ stores and Ca^2+^ release. Hureau and colleagues [22] dissociated intramuscular H^+^ and Pi in humans and reported that peripheral fatigue was more closely and consistently related to Pi than to H^+^, whereas voluntary activation was related to H^+^ but not Pi across trials. Recent in vivo rat work reported a discernible twitch force-Pi breakpoint and a more consistent force-Pi relationship than force-H^+^ relationship across stimulation conditions [23]. Westerblad [24] emphasized that force decline was well correlated with Pi accumulation above a critical level of about 15 to 20 mM, while no comparable mechanism-related correlation was observed for H^+^.

The functional Pi threshold concept indicates that force or power may be relatively preserved while phosphate-linked disturbance remains below a task-specific boundary. Once that boundary is crossed, additional Pi accumulation may be associated with a steeper negative force-Pi relationship. This threshold should not be interpreted as a universal millimolar value. It probably depends on fiber type, temperature, pH, Ca^2+^ availability, contraction mode, training status, task demands, and the performance endpoint used.

In human studies, the concept could be operationalized with piecewise or segmented regression between 31P-MRS-derived Pi indices and evoked twitch force, tetanic force, maximal voluntary force, or external power. Individual thresholds should be estimated before group averaging, because the breakpoint may differ by athlete and task. Investigators should distinguish total Pi, free Pi, and H_2_PO_4_− where feasible and should interpret 31P-MRS cautiously because it provides spatially averaged signals that may not capture fiber-type-specific or compartment-specific Pi dynamics. Threshold estimates derived from twitch force should not be transferred directly to tetanic force, voluntary force, power output, or task failure without task-specific validation.

The metabolic characteristics of severe-intensity domain exercise are consistent with this threshold interpretation. Exercise below critical power permits relative stabilization of PCr, Pi, and pH, whereas exercise above critical power produces progressive metabolic disturbance until task failure [6,7]. Intermittent exercise extends tolerance because recovery intervals permit partial restoration of PCr and reduction of Pi, with longer recovery intervals allowing greater restoration and more work above critical power [8].

The present model does not exclude acidosis. It rejects only a simple competition between Pi and H^+^. Acidosis is less convincing as a stand-alone explanation for maximal isometric force loss at physiological temperature, but H^+^ can reduce Ca^2+^ sensitivity, alter crossbridge kinetics and cycling rate, reduce power or shortening velocity, and change phosphate speciation, particularly when pH is markedly reduced and Pi is elevated. Dynamic exercise may therefore be more sensitive to H^+^-related effects than interpretations based only on maximal isometric force. Fitts [25] argued that the fatigue-inducing effects of low pH are exacerbated by elevated Pi and reduced Ca^2+^ transients, which more closely resembles in vivo fatigue than isolated low-pH conditions alone. Westerblad [26] emphasized the counterpoint: several mammalian preparations show weak or inconsistent causal relationships between intracellular acidosis and force decline, especially when acidosis is considered in isolation. The most defensible interpretation is that H^+^ and Pi interact [27–30].

### 5.2. Acid-base stress and group III/IV afferent feedback

The contractile consequences of Pi occur inside the fiber, whereas the neural consequences of intense exercise arise primarily from the interstitial and mechanical environment sensed by group III/IV afferents. High-intensity exercise is often described as being limited by lactic acid; however, this terminology is biochemically imprecise and mechanistically incomplete. Robergs and colleagues [31] argued that lactate production does not cause acidosis in the simplistic textbook sense and may retard, rather than cause, acidosis under intense exercise conditions. Brooks [32] reframed lactate as a fuel, gluconeogenic precursor, and signaling molecule rather than a waste product. Thus, lactate anion, H^+^, and broader acid-base disturbance should be distinguished.

Group III/IV afferents respond to patterns of mechanical and biochemical stimulation rather than to a single metabolite. During intense contractions, relevant stimuli are best described as interstitial and mechanical conditions, including H^+^/pH disturbance, lactate, ATP, K^+^, bradykinin, prostaglandins, vascular or interstitial chemical changes, and mechanical deformation [9,10]. Hureau and colleagues' 31P-MRS work separates Pi-linked peripheral fatigue from H^+^-linked reductions in voluntary activation, but this should be interpreted as an inference about afferent-mediated central effects rather than direct afferent-discharge evidence [22]. Blain and colleagues [11] provide the most direct whole-body support for the metabolic side of this pathway. When lower-limb group III/IV feedback was attenuated during a 5 km cycling time trial, motoneuronal output was higher and exercise-induced increases in Pi, H^+^, ADP, and lactate were greater, while PCr depletion and quadriceps twitch fatigue were also greater.

This feedback has a regulatory function. With the onset of exercise, group III/IV afferents contribute to ventilatory and cardiovascular responses that help maintain perfusion and O_2_ delivery and thereby reduce the rate of peripheral fatigue development [9,10]. The same feedback can also reduce motoneuronal output through spinal and/or supraspinal processes and contribute to reduced voluntary activation. Amann and colleagues [33] demonstrated the double-edged nature of this pathway during high-intensity constant-load cycling. Lumbar intrathecal fentanyl attenuated mu-opioid receptor-sensitive feedback from the legs. Compared with placebo, central motor drive was less inhibited, but ventilation, arterial pressure, heart rate, and oxygenation were compromised; peripheral fatigue developed faster and time to exhaustion was shorter. Hureau and colleagues [34] later controlled for the O_2_-delivery component and showed that, when locomotor muscle O_2_ delivery was preserved, afferent attenuation increased mean power by about 9%, improved completion time by about 3%, increased vastus lateralis electromyographic (EMG) activity by about 9%, and increased peripheral fatigue.

The nutritional interpretation should therefore be specific. Pharmacological afferent attenuation is not equivalent to nutritionally reducing metabolic stress. Afferent attenuation can reduce inhibitory feedback, but it can also blunt ventilatory and circulatory regulation, increase intramuscular metabolic perturbation, or alter contractile efficiency [11,17,33,34]. Nutrition interventions should be framed as approaches that reduce unnecessary metabolic or acid-base disturbance, delay afferent escalation, or improve tolerance while preserving the regulatory functions that protect muscle and systemic homeostasis.

### 5.3. Perceived effort, muscle pain, and behavioral tolerance

Perceived effort has often been treated as a direct readout of peripheral fatigue or afferent feedback. This interpretation is too simple. Effort, when narrowly defined and anchored, is closely linked to central motor command and corollary discharge [13–15]. As active muscle becomes less capable of producing force, the central nervous system may increase motor command to maintain output. Peripheral fatigue can therefore increase the central command required to maintain a given external output. Group III/IV feedback can contribute to pain, discomfort, motor regulation, and, depending on scale instructions, global perceived exertion. This explains why effort can rise when external power remains constant, but also why broad RPE should not be interpreted as effort alone.

De Morree and colleagues [14] reported that ratings of perceived effort were associated with movement-related cortical potential amplitude during dynamic elbow flexion, supporting the view that effort reflects central motor command during movement execution. Pageaux [15] argued that exercise-based research should distinguish perceived effort from broader perceived exertion. Marcora [13] provided the strongest statement of this position and argued that afferent feedback from skeletal muscle, heart, and lungs does not contribute significantly to the perception of effort during dynamic exercise when effort is narrowly defined. However, afferent attenuation can alter Borg RPE when the rating captures broader exertional sensations. In Broxterman and colleagues' intermittent leg-extensor study, fentanyl lowered CR10 RPE at task failure despite similar force-time work and greater Pi, H_2_PO_4_^-^, and ATP perturbation [17].

Muscle pain is related to, but distinct from, perceived effort. It is more directly linked to nociceptive and metaboreceptive feedback, although it is also centrally modulated [3,9,10,15,16]. During severe exercise, effort and pain may rise together because increasing central motor command and increasing afferent feedback occur in parallel. Their mechanisms and nutritional responsiveness, however, may differ. A supplement may improve performance by lowering metabolic disturbance at a given workload, allowing greater terminal disturbance, reducing pain without changing effort, or lowering perceived effort by reducing the central command required for a given output. These mechanisms are not equivalent and should not be assessed with a single post-exercise rating.

Measurement should therefore separate effort-specific ratings from pain-specific and discomfort-specific ratings and should report the anchors and instructions used. Broad RPE can be retained as a global measure, but it should not be interpreted as effort alone unless scale instructions restrict the rating to the effort required to produce the action (Tables 1 and 2).

**Table 1.** Key constructs in the Pi-afferent-effort model.

Construct	Working definition	Common measurements	Relevance to high-intensity exercise
Fatigue	Broad symptom or state interpreted through performance and perception	Self-report plus performance measures	Integrates physiology, perception, and behavior
Performance fatigability	Objective decline in force, power, work, or task output	MVC, power decrement, work completed, time to failure	Captures observable performance loss
Peripheral fatigue	Reduced contractile response at or distal to the neuromuscular junction	Potentiated twitch, evoked force, contractile properties	Linked to Pi, pH, phosphate species, Ca^2+^, crossbridge effects, and sarcolemmal excitability; not all peripheral fatigue is Pi- or H^+^-mediated
Central fatigue	Reduced voluntary neural drive from spinal or supraspinal processes	Voluntary activation, TMS, cervicomedullary stimulation	Motor-drive construct; not simply sleepiness, motivation loss, or one neurotransmitter
Group III/IV afferents	Thin-fibre sensory feedback from mechanical and chemical stimuli	Afferent attenuation; metaboreflex approaches; PECO and hypertonic-saline models interpreted cautiously	Regulate ventilation, circulation, O_2_ delivery, motor output, pain, and discomfort
Perceived effort	How hard, heavy, or strenuous the voluntary action feels	Effort-specific ratings with explicit instructions	Linked primarily to central motor command and corollary discharge when narrowly anchored
Perceived exertion	Broader exercise strain when instructions include multiple sensations	Borg or CR scales; depends on instructions	May include effort, pain, discomfort, breathlessness, heat, or fatigue sensations
Muscle pain	Nociceptive-affective sensation from active muscle	Pain scales, pain threshold, pain tolerance	Influences sensory tolerance and pacing; distinct from effort
Exercise tolerance/task failure	Work or duration sustained under a task constraint; failure may be mechanical or behavioural	Time to exhaustion, work completed, distance, voluntary termination	Final outcome of integrated boundaries

**Table 2.** Evidence status and limits of inference for key links in the Pi-afferent-effort model.

Model link	Evidence status	Direct support	Main limitation or testable prediction
Pi and contractile function	Direct mechanistic support	Reduced preparations, intact muscle, human 31P-MRS, and modeling support Pi effects on force, Ca^2+^ sensitivity, and excitation-contraction coupling.	Threshold values are task-specific and should not be treated as universal millimolar cutoffs.
H^+^ and Pi interaction	Direct and integrative support	Acid-base stress can modify Ca^2+^ sensitivity, crossbridge kinetics, power, shortening velocity, and phosphate speciation, especially when Pi is elevated.	No single H^+^ or Pi metric explains all peripheral fatigue. Studies should report pH, Pi, phosphate species where feasible, and contractile endpoints.
Group III/IV afferent feedback and motor output	Direct human support	Afferent attenuation studies show effects on motoneuronal output, voluntary activation, ventilation, circulation, metabolic perturbation, and peripheral fatigue.	Pharmacological afferent attenuation is not equivalent to nutritional reduction of metabolic or acid-base stress.
Nutrition reducing afferent escalation	Plausible inference	Nitrate, bicarbonate, beta-alanine, creatine, carbohydrate, and caffeine can alter upstream or downstream boundaries in selected tasks.	Direct evidence that these supplements reduce group III/IV afferent discharge in exercising humans is limited.
Functional Pi threshold across exercise phenotypes	Testable conceptual model	Recent threshold and severe-domain studies support task-specific Pi-force relationships and non-steady-state PCr/Pi behavior above critical power.	Requires individualized segmented analyses and validation across twitch force, tetanic force, voluntary force, power output, and task failure.
Supplement combinations	Hypothesis or conditional application	Some combinations target complementary boundaries, such as intracellular plus extracellular buffering or ATP cost plus PCr recovery.	Combination use should be justified by phenotype, evidence directness, tolerance, and athlete constraints rather than assumed additivity.

## 6. Nutritional strategies mapped to the model

The same sequence can be used to interpret supplementation. Each intervention can be evaluated according to whether it modifies phosphate-linked metabolic kinetics, acid-base stress, afferent escalation, central motor output, perceived effort, pain, substrate availability, or tolerance. This approach does not require that a supplement reduce fatigue in a general sense. Rather, it asks which boundary is changed, under which task conditions, and whether the observed benefit reflects lower strain at matched work, greater tolerance of terminal strain, or both. Table 3 classifies each strategy by its primary target and by the directness of current evidence.

**Table 3.** Nutritional strategies mapped to the Pi-afferent-effort cascade.

Strategy	Primary model target	Proposed mechanism	Best-fit phenotype	Evidence status and main limitation
Dietary nitrate	ATP cost, phosphate-linked PCr/ADP/Pi kinetics, contractile efficiency, local O_2_ matching	Nitrate-nitrite-NO pathway; lower ATP cost; improved contractile efficiency; possible Ca^2+^-related effects; improved local O_2_ delivery or O_2_ delivery-to-demand matching	Severe-intensity exercise of about 5 to 30 min; selected short-recovery intermittent high-intensity work; hypoxia	Direct evidence for economy, tolerance, and PCr perturbation in selected models; recent syntheses support mixed, small, and phenotype-dependent effects; direct afferent evidence is lacking.
Creatine monohydrate	PCr availability, ATP buffering, and between-bout recovery	Increased creatine/PCr pool; improved ATP resynthesis through creatine kinase; higher PCr availability and lower Pi/pH-disturbance carryover in repeated maximal protocols	Repeated sprint, HIIT, repeated accelerations	Direct evidence for increasing total creatine/PCr and improving repeated-bout performance; strongest rationale for repeated or intermittent tasks; body mass gain and response variability matter.
Beta-alanine	Intracellular acid-base environment and pH-dependent contractile effects	Chronic beta-alanine loading increases muscle carnosine and intracellular buffering; may indirectly alter pH-dependent Pi effects without directly lowering total Pi	0.5 to 10 min tasks, especially 1 to 4 min; repeated glycolytic work	Direct evidence for increasing muscle carnosine; performance effects are duration- and test-specific; Pi and afferent effects remain indirect.
Sodium bicarbonate	Extracellular acid-base stress, H^+^ efflux, and strong-ion/metabolic milieu	Acute or multi-day NaHCO_3_ increases blood bicarbonate, pH, and base excess; supports H^+^/lactate efflux; may affect PCr/Pi-power relationships and strong-ion distribution	30 s to 12 min high-intensity tasks, repeated bouts, combat sports; selected longer hypoxic or repeated time-trial protocols	Direct evidence for extracellular alkalosis and selected high-intensity performance benefits; GI distress, sodium load, timing, and product-specific delivery systems limit use; direct afferent evidence is lacking.
Caffeine	Arousal, motor output, effort appraisal, and pain tolerance	Adenosine antagonism, arousal, corticospinal excitability, altered effort appraisal and pain processing	Many high-intensity and endurance tasks	Direct performance support across several domains; mechanisms are partly central/perceptual; individual response, sleep, anxiety, and expectancy must be managed.
Carbohydrate	Muscle/liver glycogen, blood glucose, oral-CNS signaling	Maintains carbohydrate availability for prolonged/repeated work; mouth rinse, or small doses may alter central processing	Prolonged/repeated high-intensity exercise, team sports, tournaments, short-recovery events	Direct support for prolonged and repeated work; oral-central effects are plausible for shorter tasks; less relevant for isolated brief bouts.

### 6.1. ATP cost and phosphagen kinetics: dietary nitrate

Dietary nitrate may delay the approach to the Pi boundary by improving muscle efficiency or contractile function, reducing the ATP cost per unit force or power, blunting PCr degradation and associated ADP/Pi accumulation in some fixed-work or hypoxic models, and improving local O_2_ delivery or matching O_2_ delivery to O_2_ demand under selected conditions [35–38]. Within the Pi-afferent-effort model, nitrate is positioned upstream of afferent and perceptual escalation. If less ATP is required for a given force or power output, or if local O_2_ delivery is better matched to demand, PCr degradation and Pi accumulation may be slowed in susceptible protocols.

The nitrate-nitrite-NO pathway is mechanistically relevant to severe-intensity and hypoxic exercise because nitrite reduction is favored by lower O_2_ availability and lower pH [37,38]. Inorganic nitrate is concentrated in saliva, reduced to nitrite by oral bacteria, swallowed, and then converted to nitric oxide and related nitrogen species. Antibacterial mouthwash can blunt nitrate-to-nitrite conversion. Therefore, study controls and applied recommendations should specify mouthwash use, habitual nitrate intake, and supplement timing [37,39].

Bailey and colleagues [35] reported that nitrate-rich beetroot juice reduced the O_2_ cost of submaximal cycling and increased time to exhaustion during severe exercise. In a subsequent 31P-MRS knee-extensor study, dietary nitrate attenuated PCr degradation during low-intensity exercise, reduced the PCr and oxygen uptake slow components during high-intensity exercise, and lowered estimated ATP turnover for a given work rate [36]. These findings suggest that nitrate may alter phosphate-linked metabolic perturbation at a matched external output, rather than merely increasing motivation or masking symptoms. Jones [37] concluded that nitrate can reduce the oxygen cost of submaximal exercise and, in some circumstances, improve exercise tolerance and performance. Coggan and Peterson [40] synthesized evidence that nitrate can enhance human skeletal muscle contractile properties and proposed NO-related mechanisms involving Ca^2+^ handling and myofilament Ca^2+^ sensitivity.

Nitrate effects remain protocol- and athlete-dependent. Wylie and colleagues [41] reported a 4.2% improvement in Yo-Yo intermittent recovery performance in recreational team-sport players. Wylie and colleagues [39] later reported a 5% higher mean power across repeated 6 s sprints with 24 s recovery, driven mainly by a 7% improvement in the first six sprints, with no significant benefit for seven 30 s all-out bouts with 4 min recovery or six 60 s self-paced intervals. Husmann and colleagues [42] reported that 5 days of nitrate-rich beetroot juice improved knee-extensor time to exhaustion and reduced exercise-induced maximal voluntary torque loss, 100 Hz quadriceps twitch impairment, perceived effort, and leg muscle pain in a responder/time-matched comparison. Recent syntheses support a cautious phenotype-specific interpretation. An umbrella review of 20 systematic reviews and meta-analyses reported benefits for several outcomes, including time-to-exhaustion tasks, total distance covered, muscular endurance, peak power output, and time to peak power output, but also found equivocal effects for time-trial performance and substantial methodological limitations in existing reviews [43]. A systematic review and meta-analysis of randomized controlled trials focused on short-duration high-intensity exercise reported small positive effects for time to peak power, mean power, and total distance covered for the Yo-Yo Intermittent Recovery Test Level 1, while emphasizing protocol heterogeneity and the need for mechanism-focused research [44]. The available evidence does not show that nitrate directly lowers intramyocellular Pi or group III/IV afferent discharge in all tasks. Thus, the afferent component should be treated as an upstream inference rather than a demonstrated neural effect.

### 6.2. ATP buffering and between-bout recovery: creatine monohydrate

Creatine monohydrate may delay peripheral fatigue most plausibly in repeated or intermittent high-intensity exercise by increasing the intramuscular total creatine pool, including PCr, and thereby expanding substrate availability for the creatine kinase reaction [45,46]. Within the Pi-afferent-effort model, creatine is best interpreted as a phosphagen availability, ATP-buffering, and between-bout recovery intervention, rather than as a direct central or perceptual aid. Its relevance to Pi is indirect. Creatine may reduce the residual PCr depletion, Pi accumulation, and pH disturbance carried into subsequent bouts, but it should not be described as a universal method for lowering Pi during every high-intensity task [47,48].

Casey and colleagues [46] showed that 5 days of creatine ingestion increased muscle total creatine, increased work production during two 30 s maximal cycling bouts, and reduced cumulative ATP loss despite greater work output. Resting PCr increased in type I and type II fibers, and changes in type II fiber PCr availability were linked to PCr degradation and work production. Yquel and colleagues [47] provided the most direct evidence that creatine can influence PCr resynthesis and Pi carryover during intermittent maximal exercise. After 6 days of creatine loading, repeated maximal plantar-flexion power increased by approximately 5% from bouts 3 to 7. During recovery, PCr resynthesis increased, and the higher PCr after 30 s of recovery was accompanied by lower Pi accumulation and higher pH. This should be interpreted primarily as a between-bout recovery mechanism: creatine may improve PCr resynthesis and reduce Pi carryover during recovery, but during maximal or open-ended exercise it may also permit greater ATP turnover and therefore greater absolute Pi accumulation before task failure.

Jones and colleagues [48] used 31P-MRS during moderate- and heavy-intensity knee-extensor exercise and found that creatine supplementation increased the resting PCr-to-ATP ratio and slowed PCr kinetics during the moderate on- and off-transients and the heavy on-transient, whereas heavy-exercise recovery kinetics were not significantly changed. These findings indicate that creatine loading can alter PCr dynamics through increased metabolic capacitance, but they should not be cited as evidence that creatine universally accelerates PCr recovery. The direction of the kinetic effect depends on the exercise model [47,48].

Nitrate and creatine are mechanistically complementary. Nitrate may reduce ATP cost per unit force or power or improve contractile function in selected conditions, whereas creatine may increase phosphagen availability and support between-bout ATP resynthesis. The model therefore predicts possible complementarity for intermittent severe-intensity exercise, repeated sprint cycling, repeated accelerations, and combat sport flurries. Forbes and colleagues [49] noted that creatine has potential benefits for HIIT contexts, but longer-term studies combining creatine with interval training and integrated mechanistic endpoints remain limited. Thus, nitrate plus creatine should be treated as a testable hypothesis rather than an established combined-supplement recommendation.

### 6.3. Acid-base regulation: beta-alanine and sodium bicarbonate

Current evidence does not support beta-alanine as a primary Pi-lowering or PCr-restoring intervention. Its established effect is to increase skeletal-muscle carnosine after chronic loading and thereby improve intracellular physicochemical buffering over the physiological pH range. Additional roles for carnosine in Ca^2+^ sensitivity or antioxidant chemistry remain plausible but are less central to the performance rationale in high-intensity exercise [50]. The ISSN beta-alanine position stand concluded that 4 to 6 g/day for about 4 weeks significantly augments muscle carnosine and that daily supplementation for at least 2 to 4 weeks may improve exercise performance, with the most consistent evidence in high-intensity tasks lasting about 1 to 4 min [50].

Derave and colleagues [51] showed that 4 to 5 weeks of beta-alanine loading increased carnosine in the soleus by 47% and gastrocnemius by 37% in sprint-trained men and attenuated fatigue during repeated maximal isokinetic knee-extension bouts. The study did not improve isometric endurance or 400 m race time; therefore, it supports task-specific fatigue resistance rather than a broad sprint-race claim. Meta-analytic evidence also supports duration specificity. Hobson and colleagues [52] estimated a median 2.85% improvement versus placebo, with significant effects for exercise lasting 60 to 240 s and more than 240 s, but not for efforts shorter than 60 s. Saunders and colleagues [53] reported a small significant overall effect size of 0.18, identified exercise duration as the main moderator, and supported the 0.5 to 10 min range, with larger effects for exercise-capacity tests than fixed-end performance tests.

Beta-alanine may not reduce total Pi, but by increasing carnosine-mediated intracellular buffering it may alter the pH-dependent environment in which Pi depresses force, shortening velocity, and power. Any afferent implication is indirect. Beta-alanine has not been shown to make group III/IV afferents sense less H_2_PO_4_^-^ or to directly suppress afferent discharge. A defensible prediction is that, in work-matched high-glycolytic tasks, beta-alanine might reduce the intracellular acid-base component of peripheral fatigue and secondarily reduce pain or broad exertional sensations. This prediction requires direct testing with 31P-MRS, pH kinetics, contractile function, effort-specific ratings, and pain outcomes [21,50,53].

Sodium bicarbonate acts primarily on the extracellular pH side of the cascade. It may reduce acid-base stress by increasing extracellular buffering capacity, raising blood bicarbonate, pH, and base excess, and increasing the transmembrane gradient that supports H^+^ and lactate efflux from active muscle, most likely through monocarboxylate transporters [54]. Mechanistic reviews also emphasize effects on the PCr/Pi-power relationship, glycolytic intermediates, intra- and extracellular strong-ion distribution, membrane excitability, rate of force development, and motor pathways [54,55]. Any effect on group III/IV feedback should be treated as a plausible indirect mechanism rather than a directly demonstrated human supplementation effect.

The ISSN position stand concluded that sodium bicarbonate supplementation improves performance in muscular endurance activities, combat sports, and high-intensity cycling, running, swimming, and rowing, with effects mostly established for high-intensity tasks lasting approximately 30 s to 12 min [54]. This duration range overlaps with exercise phenotypes in which H^+^ stress, Pi accumulation, pain, effort, and expected afferent stimulation escalate rapidly, but it should not be treated as a hard physiological cutoff. Recent hydrogel mini-tablet studies [56,57] reported low-GI, product-specific benefits in repeated 4 km cycling and in a 40 km cycling time trial performed in acute normobaric hypoxia. These findings extend the context in which bicarbonate may be useful, but they should not be generalized to all longer events without further testing [56,57].

Beta-alanine and bicarbonate are mechanistically complementary because beta-alanine targets intracellular buffering through carnosine, whereas bicarbonate targets extracellular buffering, alkalosis, and H^+^ efflux [50,54]. Tobias and colleagues [58] provided proof-of-concept evidence in judo and jiu-jitsu athletes: 6.4 g/day beta-alanine for 4 weeks and 0.5 g/kg/day sodium bicarbonate for 7 days each increased total work during repeated upper-body Wingate bouts by about 7% and 8%, respectively, whereas co-ingestion increased total work by about 14% and was the only condition associated with lower perceived exertion. Curran-Bowen and colleagues [59] later reported that combined beta-alanine and sodium bicarbonate improved exercise outcomes in a 10-study, 243-participant meta-analysis. Because their meta-regression did not detect significant differences among supplement types, the combination is plausible when both intracellular and extracellular buffering are challenged, but it should not be framed as uniformly additive or as overriding the broader isolated-supplement evidence for either supplement [50,52–54,59].

### 6.4. Central, perceptual, and substrate support: caffeine and carbohydrate

Caffeine may improve high-intensity performance primarily through adenosine receptor antagonism, arousal, vigilance, corticospinal excitability, motor output, perceived effort, pain modulation and tolerance [60–62]. These mechanisms should be distinguished from older central-fatigue neurotransmitter hypotheses. Serotonergic, dopaminergic, and noradrenergic models are relevant to arousal, motivation, thermoregulation, and prolonged exercise, but they are not sufficient as a stand-alone explanation for acute high-intensity exercise failure [4,60]. Caffeine should not be conceptualized primarily as a Pi-lowering or acid-base intervention. In the Pi-afferent-effort model, caffeine acts mainly downstream of peripheral metabolic disturbance and afferent input, although peripheral contractile and ion-handling effects may also occur.

The ISSN caffeine position stand concluded that caffeine can acutely enhance several aspects of exercise performance, with consistent effects at approximately 3 to 6 mg/kg body mass and important individual differences in response [60]. Grgic and colleagues' umbrella review found ergogenic effects across aerobic endurance, strength, muscular endurance, power, jumping, and speed outcomes, although evidence quality varied across domains [61]. Bowtell and colleagues [62] provided an important example for the present model. In repeated intense leg-extensor exercise, caffeine increased total exercise time, but later exercise sets were associated with lower PCr and lower pH. Thus, performance improved despite greater metabolic disturbance. This pattern suggests that caffeine may shift arousal, motor output, effort appraisal, pain tolerance, or willingness to continue rather than simply reduce the peripheral disturbance required to fail. Causality remains unresolved because greater terminal disturbance can also increase afferent inhibitory input.

Carbohydrate is most relevant when high-intensity exercise is prolonged, intermittent, or repeated enough that muscle glycogen, liver glycogen, blood glucose, central nervous system function, or late-session skill and effort responses can become limiting. In the Pi-afferent-effort model, carbohydrate is not an acute Pi-lowering intervention for an isolated 30 s sprint. Its primary role is to preserve carbohydrate availability for key high-quality or competitive work, support between-session glycogen restoration, and, in shorter high-intensity tasks, potentially influence performance through oral carbohydrate sensing and central mechanisms. Conversely, deliberately low carbohydrate availability can be used as a periodized training stimulus in selected contexts, but it should not be confused with the fuelling strategy most appropriate for key high-intensity performance sessions [49,63,64].

Thomas and colleagues [63] and Burke and colleagues [64] framed carbohydrate targets around training load, event demands, and the goal of high versus low carbohydrate availability. Carbohydrate is usually unnecessary for brief exercise shorter than 45 min. Small amounts or mouth rinse may help sustained high-intensity exercise of approximately 45 to 75 min. A dose of 30 to 60 g/h is commonly recommended for 1 to 2.5 h of endurance or stop-start exercise, and up to approximately 90 g/h is used for events beyond about 2.5 to 3 h when multiple transportable carbohydrates and gut tolerance permit [63–66]. Carbohydrate mouth-rinse and small-dose effects are relevant to the effort side of the model because they show that carbohydrate can improve performance in some 30 to 75 min high-intensity tasks without a large metabolic substrate contribution. The most cautious interpretation is that oral carbohydrate sensing can influence brain activity, reward or perceptual processing, or motor output; the exact receptors and pathways remain incompletely defined [65,66].

## 7. Exercise phenotype-specific application

**Figure 1.**
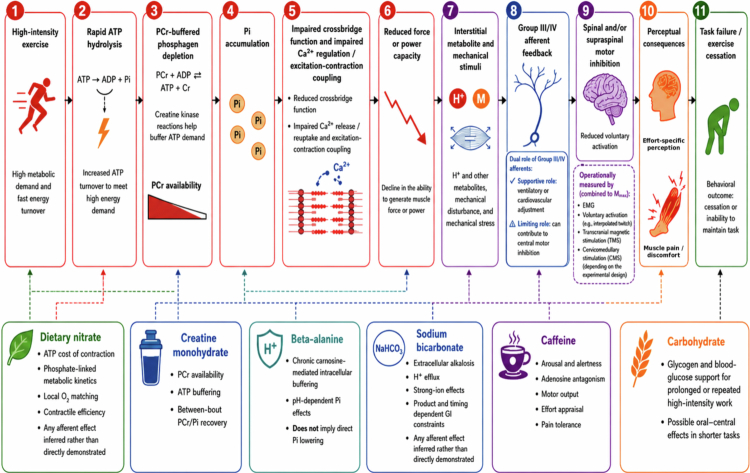
The Pi-afferent-effort model of high-intensity exercise task failure. The model shows the proposed cascade from ATP hydrolysis, PCr-buffered phosphagen depletion, and Pi accumulation to impaired crossbridge function and Ca^2+^ regulation, reduced force capacity, interstitial metabolite/mechanical group III/IV afferent stimuli, afferent feedback with dual support and limitation roles, spinal and/or supraspinal motor inhibition, reduced voluntary activation, effort-specific perception, muscle pain/discomfort, and task failure. Nutritional strategies are mapped to proposed mechanistic targets: nitrate to ATP cost, phosphate-linked metabolic kinetics, local O_2_ matching, and contractile efficiency; creatine to PCr availability and between-bout PCr/Pi recovery; beta-alanine to chronic carnosine-mediated intracellular buffering and pH-dependent Pi effects; sodium bicarbonate to extracellular alkalosis and H^+^ efflux; caffeine to arousal, motor output, effort appraisal, and pain tolerance; and carbohydrate to glycogen/blood-glucose support and possible oral-central effects. Broad RPE may include effort, pain, breathlessness, discomfort, and fatigue sensations depending on scale instructions; therefore, effort-specific perception and muscle pain/discomfort are separated in the model.

**Figure 2.**
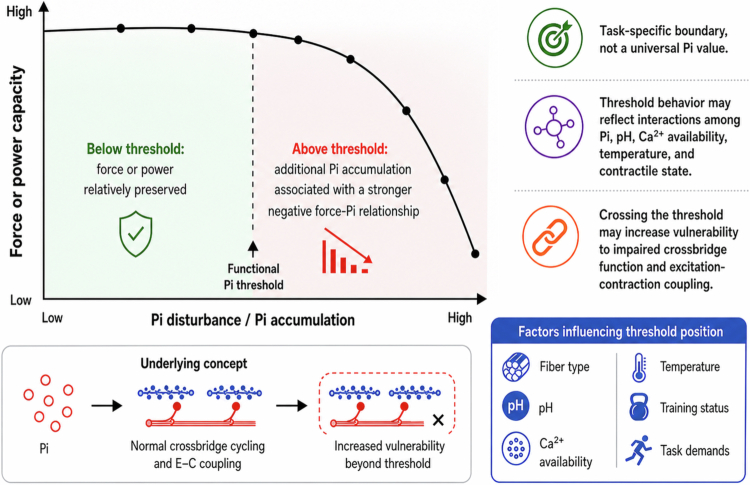
Functional Pi threshold concept. Force or power capacity may be relatively preserved below a task-specific Pi disturbance. After threshold crossing, additional Pi accumulation is associated with a stronger negative force-Pi relationship. The threshold is not proposed as a universal value, but as a functional boundary influenced by fiber type, pH, Ca^2+^ availability, temperature, training status, and task demands.

Because these mechanisms are weighted differently across tasks, supplement selection should be organized by exercise phenotype rather than by sport name alone. The categories below identify likely starting points, conditional options, and hypotheses; they are not stacking prescriptions. For continuous 1 to 4 min efforts, acid-base strategies are the most logical starting point because glycolytic flux, Pi accumulation, decreasing pH, effort, and pain rise rapidly. Beta-alanine and sodium bicarbonate are primary considerations when intracellular and extracellular buffering are plausible constraints. Caffeine is conditional when arousal, pain tolerance, or motor output are limiting. Nitrate is hypothesis-generating or role-specific for a single 1 to 4 min bout unless the task has a high type II fiber contribution, repeated-start features, hypoxic stress, or sufficient duration for efficiency effects to matter. Creatine is most relevant when the event or training context includes repeated starts, accelerations, or adaptation goals rather than a single continuous bout (Figures 1 and 2).

**Figure 3.**
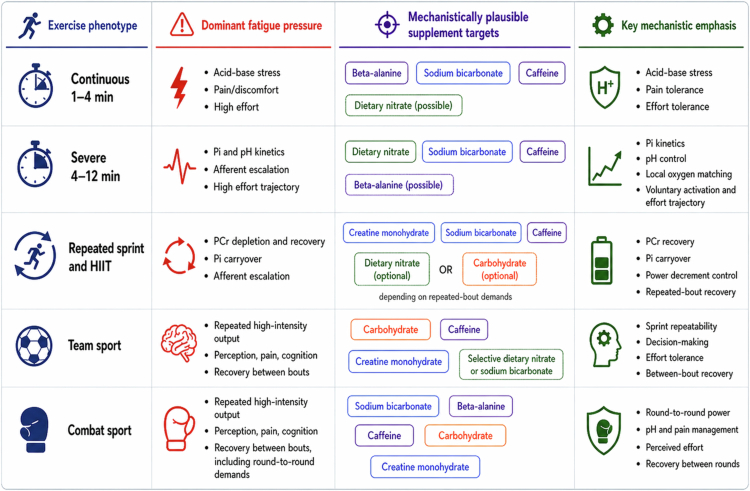
Nutritional targets by exercise phenotype. The matrix links exercise demands to dominant fatigue pressures and supplement targets. Continuous 1 to 4 min efforts emphasize acid-base stress; severe 4 to 12 min efforts emphasize Pi and pH kinetics; repeated sprint and HIIT emphasize PCr recovery and Pi carryover; team and combat sports emphasize repeated high-intensity output, perception, pain, cognition, and recovery between bouts. Longer 12 to 40 min severe-domain tasks are treated as a continuous severe-domain subcategory rather than a separate figure row and require added attention to pacing, oxygen uptake kinetics, and carbohydrate availability. Targets should be interpreted with the evidence-directness categories in Tables 3 and 4 and are not intended as universal supplement stacks.

For continuous 4 to 12 min severe-intensity efforts, PCr decreases, Pi increases, pH decreases, the oxygen uptake slow component develops, and group III/IV afferent stimulation would be expected to escalate. Nitrate is mechanistically attractive because it may reduce ATP cost and attenuate PCr/ADP/Pi perturbation at matched output, but recent syntheses support small or mixed effects depending on outcome and population [37,43,44]. Bicarbonate may help when acid-base stress is high. Caffeine may allow greater terminal PCr depletion or acidosis in some protocols, rather than lowering metabolic disturbance. Longer severe-domain efforts of approximately 12 to 40 min should be treated as a continuous severe-domain subcategory rather than a separate phenotype in Figure 3. In those tasks, sustained metabolic instability, W-prime expenditure, oxygen uptake kinetics, pacing, effort trajectory, and carbohydrate availability become increasingly important [63–66].

**Table 4.** Exercise phenotype-specific supplement logic.

Exercise phenotype	Dominant fatigue pressure	Mechanistically plausible strategies	Key measurement targets
Continuous 1 to 4 min	Acid-base stress, Pi rise, pain, high effort	Primary: beta-alanine after chronic loading and sodium bicarbonate after individualized acute or multi-day dosing. Conditional: caffeine. Hypothesis/role-specific: nitrate when type II fiber demand, hypoxia, repeated-start features, or duration support the mechanism; creatine mainly for repeated starts or training adaptation.	pH kinetics, Pi, pain-specific ratings, effort-specific ratings, evoked force
Continuous 4 to 12 min	Severe-domain instability, Pi and pH kinetics, afferent escalation; longer 12 to 40 min variants add pacing and carbohydrate-availability pressures	Primary/conditional: nitrate and caffeine depending on athlete and task. Conditional: sodium bicarbonate when acid-base stress is high; beta-alanine when task duration and acid load fit the evidence window; carbohydrate when the event follows prior work or includes repeated competition.	PCr/Pi kinetics, pH, voluntary activation, effort, pain, oxygen uptake kinetics, pacing
Repeated sprint and HIIT	PCr depletion and recovery, Pi carryover, afferent escalation, power decrement	Primary: creatine. Conditional: sodium bicarbonate, caffeine, carbohydrate for long sessions, selected nitrate for short-recovery/high type II protocols, and beta-alanine when repeated glycolytic bouts are sufficiently prolonged.	PCr recovery, Pi area above threshold, power decrement, normalized EMG/Mmax, voluntary activation, effort-specific ratings, pain ratings
Team sport	Repeated bursts, glycogen, cognition, skill, recovery between bouts	Foundational: carbohydrate and caffeine. Conditional: creatine. Role-specific: nitrate, bicarbonate, or beta-alanine when athlete role and high-intensity density fit the mechanism.	Sprint repeatability, decision-making, effort, pain, GI tolerance, late-session skill
Combat sport	Glycolytic flurries, pain, arousal, recovery between rounds	Primary/conditional: beta-alanine and sodium bicarbonate when acid-base stress is prominent. Conditional: caffeine, carbohydrate for tournaments, creatine when body mass permits. Optional/hypothesis-generating: nitrate in selected intermittent severe-intensity profiles.	Round-to-round power, pH, pain, perceived effort, GI tolerance, body mass

Repeated sprint and HIIT tasks are especially well suited to the model. Each bout perturbs PCr, Pi, and pH, and each recovery period partially restores homeostasis. Creatine is a primary consideration when the aim is to improve phosphagen availability and between-bout recovery. Nitrate is conditional and should be reserved for short-recovery protocols, high type II fiber demand, or athletes with demonstrated responsiveness; it should not be assumed effective for all sprint durations or recovery structures [39,44]. Bicarbonate and beta-alanine are conditional when repeated bouts are sufficiently glycolytic or long enough to produce substantial acid-base stress. Caffeine may improve motor output and tolerance, but it may also permit greater terminal disturbance. Carbohydrate is relevant when the session is long, repeated over a day, or intended as a high-quality HIIT session rather than a deliberately low-carbohydrate adaptation stimulus [49,63].

Team sports combine repeated accelerations, incomplete recovery, skill, cognitive load, and contextual decision-making. No single fatigue boundary dominates. Carbohydrate and caffeine are often foundational because carbohydrate supports substrate availability and may help preserve late-session skill or perceptual outcomes, whereas caffeine supports vigilance, drive, and tolerance [60,63,65,66]. Creatine is conditional when repeated power output is a priority and body mass changes are acceptable. Nitrate or bicarbonate may be useful for specific roles, phases, or athletes, but nitrate evidence is strongest for recreational team-sport players in Yo-Yo IR1 and selected short-recovery sprint protocols, not for every team-sport context [39,41,43,44].

Combat sports involve repeated glycolytic bursts, isometric and dynamic contractions, psychological stress, pain, arousal, and recovery between rounds. Bicarbonate and beta-alanine are strong candidates when acid-base stress is prominent, and their combination has proof-of-concept evidence in judo and jiu-jitsu upper-body Wingate bouts [54,58,59]. Applied use must still be tested against weight-class, sodium-load, gastrointestinal, and timing constraints. Caffeine may improve arousal, drive, and tolerance, but dose must be balanced against anxiety, tremor, and sleep. Carbohydrate is important for tournaments and long training sessions, especially when multiple bouts or short recovery windows require glycogen restoration [63,65]. Creatine may support repeated high-force efforts, but body mass implications matter in weight-class contexts.

To translate these phenotype-specific principles into applied interpretation, Supplementary File 1 provides three hypothetical case scenarios based on the Pi-afferent-effort model: one continuous high-intensity exercise scenario, one repeated sprint sport scenario, and one combat sport scenario. Each scenario links the dominant fatigue pressures of the task to primary, conditional, optional, or hypothesis-generating supplementation strategies, timing before competition, and key practical constraints. These examples are model-based decision aids rather than prescriptive dosing templates and should be individualized according to athlete characteristics, training status, supplement tolerance, body-mass constraints, medical history, anti-doping requirements, and event rules (Box 1).

**Box 1.** Testable predictions of the Pi-afferent-effort model.

Prediction	Implication
1	Nitrate may delay approach to a functional Pi threshold by reducing ATP cost and phosphate-linked perturbation in susceptible tasks, whereas creatine may delay it by improving PCr availability and recovery. Neither is expected primarily to change the force-Pi slope once the threshold is crossed.
2	Beta-alanine and sodium bicarbonate are not primary total-Pi-lowering interventions. They may modify pH-dependent Pi effects and acid-base-related sensations at matched work, but direct evidence for reduced group III/IV afferent discharge is lacking.
3	Caffeine may improve performance while permitting greater PCr depletion and pH decline, and potentially greater Pi where measured, indicating increased tolerance or motor output rather than reduced metabolic disturbance.
4	Nitrate plus creatine is an untested combined-supplement prediction for intermittent high-intensity exercise because the combination targets ATP cost or contractile efficiency plus PCr/Pi recovery.
5	Beta-alanine plus sodium bicarbonate may be most effective when the task produces substantial intracellular and extracellular acid-base stress, but available evidence does not justify assuming uniform additivity.
6	The optimal supplement strategy depends on exercise phenotype, not sport name alone.

## 8. Future research agenda

A major limitation of the current literature is that many supplement studies measure performance without measuring the pathway through which performance changed. A trial showing improved time to exhaustion after nitrate, bicarbonate, beta-alanine, creatine, or caffeine does not reveal whether the supplement delayed Pi accumulation, altered pH, reduced peripheral fatigue, changed voluntary activation, lowered perceived effort, reduced pain, or increased tolerance of greater terminal disturbance. Future trials should therefore combine work-matched and performance-maximized designs. Work-matched trials can determine whether a supplement reduces physiological or perceptual strain at the same external output. Performance-maximized trials can determine whether a supplement allows greater work before similar or greater terminal strain.

Core outcomes should include performance under both work-matched and performance-maximised conditions; 31P-MRS for PCr, Pi, and pH kinetics when feasible; evoked twitch force for peripheral fatigue; MVC and voluntary activation for voluntary neural drive; blood gases, bicarbonate, lactate, and pH when acid-base interventions are tested; and separately anchored ratings of perceived effort, muscle pain, discomfort, arousal, and willingness to continue. Broad RPE can be retained, but it should not replace effort-specific and pain-specific ratings.

Advanced outcomes, when resources permit, should include phosphate speciation, normalized EMG/M-wave, transcranial magnetic stimulation, cervicomedullary stimulation, near-infrared spectroscopy, post-exercise circulatory occlusion designs, and afferent attenuation or manipulation models. These methods are not required for every trial, but they are needed to determine whether a supplement changes peripheral metabolic kinetics, contractile function, voluntary activation, afferent-related sensations, or tolerance of terminal strain.

Several variables are especially important: time to functional Pi threshold; Pi accumulation rate; Pi area above threshold; PCr depletion and recovery time constant; pH kinetics; force-Pi slope after threshold crossing; voluntary activation trajectory; effort and pain trajectories; and terminal metabolic disturbance at task failure. For nitrate, future trials should measure plasma nitrate/nitrite, oral nitrate-reducing controls, 31P-MRS-derived PCr/Pi/pH kinetics, evoked contractile function, effort, pain, and terminal metabolic disturbance in both work-matched and performance-maximized designs. For beta-alanine, trials should document baseline and post-supplementation muscle carnosine when feasible, total dose, duration, adherence, and whether performance changes align with pH and phosphate-speciation kinetics [50,53]. For bicarbonate, trials should report blood bicarbonate, pH or base excess, individual time-to-peak, sodium load, gastrointestinal symptom burden, delivery system, and whether benefits reflect reduced work-matched strain or greater performance-maximized terminal disturbance [54–57,67].

Blain and colleagues [11] provide the key whole-body evidence that afferent attenuation increases Pi, H^+^, ADP, lactate, PCr depletion, and twitch fatigue during cycling. Broxterman and colleagues [68] showed that all-out exercise can produce an increasing ATP cost of contraction and can link peripheral fatigue to intramuscular pH and phosphate species. Their later work suggests an indirect role for group III/IV afferents in maintaining contractile efficiency during exercise, in addition to constraining metabolic perturbation [17]. Hureau and colleagues' repeated sprint work supports the concept that indices of central motor drive and power output may level off as peripheral fatigue reaches a task-specific threshold [69]. Modeling approaches, including Pi double-threshold concepts, may help generate predictions about how training and supplementation alter the approach to task failure [70].

## 9. Practical applications and safety

### 9.1. Phenotype-specific application

The Pi-afferent-effort model supports targeted, phenotype-specific supplement use. For severe-intensity continuous exercise, nitrate and caffeine may be relevant, with nitrate most defensible in 5 to 30 min severe-intensity or tolerance-type tasks and less certain in highly trained or lower-intensity longer events. Bicarbonate or beta-alanine can be added when acid-base stress is large, but beta-alanine requires weeks of loading and bicarbonate requires individual timing and gastrointestinal-tolerability testing. For repeated sprint and HIIT, creatine, selected nitrate, bicarbonate, beta-alanine, and caffeine may each be useful, but for different reasons and with protocol-specific evidence. For combat sports, beta-alanine and bicarbonate are attractive for repeated glycolytic work, with co-supplementation plausible when both intracellular and extracellular buffering are challenged. For team sports, carbohydrate and caffeine are often foundational, with creatine, nitrate, beta-alanine, and bicarbonate considered according to role, training phase, high-intensity density, and tolerance [54,58–60,63–66]. Applied examples of these decision rules are provided in Supplementary File 1, which illustrates how supplement selection and timing may differ across continuous high-intensity exercise, repeated sprint sport, and combat sport contexts.

### 9.2. Moderator and responder considerations

Responder status should be interpreted according to both athlete characteristics and task demands. Nitrate responses may be smaller or less consistent in highly trained endurance athletes and may depend on habitual nitrate intake, oral nitrate-reducing capacity, mouthwash use, dose, and supplementation duration [37,43,44]. Creatine response depends partly on baseline muscle creatine, dietary pattern, dosing adherence, and whether body mass gain is acceptable [45]. Beta-alanine response depends on cumulative dose, baseline carnosine, loading duration, paraesthesia management, and task duration [50,53]. Bicarbonate response depends on blood bicarbonate response, individual time-to-peak, sodium load, delivery system, and gastrointestinal tolerance [54,56,57,67]. Caffeine response depends on dose, habituation, expectancy, anxiety, sleep, and event timing [60,61]. Carbohydrate strategy depends on event duration, prior fuelling, gastrointestinal tolerance, recovery window, and whether high or deliberately lower carbohydrate availability is desired [63–66].

### 9.3. Tolerability and contraindications

Safety and practicality should also be considered. Bicarbonate can cause gastrointestinal symptoms and has sodium-load implications. Athletes should test dose, delivery system, and timing before competition rather than assume that alkalosis will translate into performance [54,56,67]. Caffeine can worsen anxiety, sleep, tremor, and palpitations in susceptible athletes. Creatine can increase body mass and, although extensive reviews generally describe creatine monohydrate as safe and well tolerated in healthy individuals when used appropriately, mass gain can matter in weight-sensitive or weight-class sports [45]. Nitrate response can be influenced by habitual diet, oral nitrate-reducing capacity, and oral antibacterial mouthwash. Practical protocols commonly use approximately 5 to 9 mmol nitrate/day for 1 to 15 days, with acute ingestion timed so that elevated plasma nitrite is likely during exercise, typically about 2 to 3 h after a bolus. There is no clear evidence that higher intakes provide additional benefit [37,39]. Beta-alanine requires chronic loading and may cause paraesthesia, although divided lower doses or sustained-release formulations can attenuate this side effect [50]. Carbohydrate strategies should consider event duration, gastrointestinal tolerance, recovery window, and whether high or deliberately lower carbohydrate availability is desired [49,63,66].

### 9.4. Supplement quality and anti-doping risk

Supplement quality is also important. Athletes subject to anti-doping rules should use third-party tested products, avoid unverified blends, and work within sport-governing-body guidance when possible [71]. The model is mechanistic and should not be used to justify maximal stacking. Supplement combinations should be justified by complementary targets, compatibility, tolerability, and the athlete's event demands. They should also be tested in training before competition because gastrointestinal symptoms, sleep disruption, sodium load, body mass changes, or anxiety can offset a mechanistically plausible benefit.

## 10. Conclusions

High-intensity exercise task failure is best interpreted as the convergence of intramuscular, neural, perceptual, and behavioral boundaries. The Pi-afferent-effort model proposes that rapid ATP turnover increases Pi, while PCr breakdown through the creatine kinase reaction buffers ATP availability. Rising Pi contributes to peripheral force impairment once a functional threshold is crossed. Interstitial acid-base and metabolite/mechanical stimuli activate group III/IV afferent feedback, which supports cardiorespiratory regulation while also constraining motoneuronal output and central motor drive depending on task, sensory state, and O_2_-delivery context [11,33,34]. Perceived effort and muscle pain influence task disengagement, but they are not simple metabolite readouts. Effort is most closely tied to central motor command, whereas pain and discomfort are more closely tied to nociceptive-affective processing of afferent feedback and context.

The practical implication is that supplementation should be matched to the dominant fatigue pressure of the task. Nitrate and creatine are most logically interpreted as interventions affecting ATP cost, PCr availability, and phosphate-linked kinetics; beta-alanine and bicarbonate as interventions affecting intracellular and extracellular acid-base stress; caffeine as a central and perceptual-tolerance intervention; and carbohydrate as a substrate and oral-central support strategy for prolonged or repeated high-intensity work. Future studies should test these claims with integrated metabolic, neuromuscular, perceptual, and performance outcomes rather than performance alone.

## Supplementary Material

Supplementary Material JISSN_Supplemental_Case_Scenarios.docx

## Data Availability

Not applicable. No new datasets were generated or analyzed for this narrative review.
