# Supplementary material for: Delaying task failure in high-intensity exercise: a Pi-afferent-effort framework for targeted sports nutrition
Source: J Int Soc Sports Nutr. 2026 Jul 30;23(1):2711031. doi: 10.1080/15502783.2026.2711031 (PMC13425531; doi:10.1080/15502783.2026.2711031)
Supplement: Supplementary Material — JISSN_Supplemental_Case_Scenarios.docx [file RSSN_A_2711031_SM5797.docx]

**Supplementary File 1**

**Applied Case Scenarios for the Pi-Afferent-Effort Framework**

*Theoretical Supplement Selection and Timing for High-Intensity Exercise Phenotypes*

PURPOSE AND SCOPE

This supplementary file provides three applied case scenarios to illustrate how the Pi-afferent-effort model can be translated into theoretical decisions about supplement selection and timing. The cases are intentionally phenotype-based rather than product-based. For each scenario, the dominant fatigue pressure, the most plausible supplement targets, and a competition-timing sequence are identified. The examples are provided for manuscript illustration and hypothesis generation and should not be interpreted as prescriptive individual nutrition plans.

Each case should be interpreted as a decision example rather than a dosing template. The hierarchy uses the terms primary, conditional, optional, and hypothesis-generating to reduce the likelihood that the examples are interpreted as maximal supplement stacks.

All strategies should be tested in training or simulated competition before use in competition. Athletes should use third-party tested products and should account for body mass, gastrointestinal tolerance, sleep, anxiety, medications, medical history, and anti-doping rules [71].

TABLE S1

Summary of theoretical supplement strategies by case scenario

| Case scenario | Best-fit theoretical strategy and evidence status | Principal implementation caveat |
| --- | --- | --- |
| Continuous high-intensity exercise | Primary: beta-alanine, sodium bicarbonate, and caffeine when acid-base stress and tolerance are dominant. Conditional: dietary nitrate when the task resembles severe-domain continuous exercise, has a high type II fiber contribution, or lasts long enough for efficiency and phosphate-linked kinetic effects to be relevant. Carbohydrate is most relevant when the event follows prior work or includes multiple heats. | The strongest rationale is acid-base stress, effort tolerance, and pain tolerance. Bicarbonate timing and gastrointestinal tolerance must be individualized. Nitrate is plausible but is less direct for a single, very short continuous event. |
| Repeated sprint sport | Primary: creatine, carbohydrate, and caffeine. Conditional: selected nitrate use. Conditional: sodium bicarbonate and beta-alanine when repeated-sprint density, bout duration, or tournament format creates substantial glycolytic and acid-base stress. | The strategy should support PCr recovery, reduce phosphate-linked carryover, preserve carbohydrate availability, and improve arousal and tolerance without adding gastrointestinal symptoms. |
| Combat sport | Primary/conditional: beta-alanine plus sodium bicarbonate, caffeine, and carbohydrate. Conditional: creatine when body mass constraints allow. Optional/hypothesis-generating: nitrate when intermittent flurries and incomplete recovery make local efficiency or contractile effects plausible. | Weight-class constraints are central. Sodium load, water shifts, creatine-associated mass gain, caffeine anxiety, and bicarbonate gastrointestinal symptoms can offset theoretical benefits. |

Case Scenario 1 Continuous High-Intensity Exercise

**Decision status:** This scenario is intended as a model-based example, not an individualized prescription. The full sequence should be tested in training or simulated competition before use in competition.

**Scenario:** A trained athlete is preparing for a single continuous high-intensity event lasting approximately 2 to 6 minutes. Examples include an 800 m to 1500 m running analogue, middle-distance track cycling, a short rowing effort, or a severe-intensity laboratory time trial.

**Dominant model pressure:** This event is expected to produce rapid ATP turnover, PCr depletion, Pi accumulation, decreasing pH, rising perceived effort, and increasing muscle pain. The most relevant nutritional targets are intracellular buffering, extracellular buffering and H+ efflux, central arousal and tolerance, and, in some cases, reduced ATP cost or phosphate-linked perturbation.

**THEORETICAL BEST-FIT SUPPLEMENT HIERARCHY**

- Beta-alanine is the long-term strategy for intracellular buffering. It is most defensible when the event or decisive surge falls within the high-glycolytic 0.5 to 10 minute range, with the strongest logic for 1 to 4 minute events [50-53].
- Sodium bicarbonate is the acute or short-loading strategy for extracellular buffering. It is most relevant when acid-base stress is expected to be high and gastrointestinal tolerance has been confirmed [54,55].
- Caffeine is the acute strategy for arousal, motor output, effort appraisal, and pain tolerance. The dose should be individualized because higher doses can worsen anxiety, tremor, sleep, or gastrointestinal symptoms [60-62].
- Dietary nitrate is a conditional addition. It is most plausible when the continuous effort is long enough for efficiency effects to matter, when severe-domain instability is prominent, or when the event involves hypoxic or type II fiber-dependent work [35-42].
- Carbohydrate is usually not the central acute ergogenic aid for an isolated 2 to 6 minute effort. Pre-event carbohydrate availability is important, however, if the event occurs after prior training, repeated heats, or a long warm-up [63-66].

TABLE S2

Competition-timing plan for continuous high-intensity exercise

| Timing | Suggested strategy | Mechanistic purpose |
| --- | --- | --- |
| 4 to 10 weeks before competition | Begin beta-alanine at 4 to 6 g/day, divided across the day or provided as sustained-release dosing if paresthesia is problematic. Begin caffeine and bicarbonate tolerance trials in race-specific sessions. | Increase muscle carnosine and intracellular buffering. Determine whether caffeine improves drive and tolerance without excessive anxiety. Determine bicarbonate timing and gastrointestinal tolerance. |
| 1 to 2 weeks before competition | Finalize the stack and do not introduce new supplements. If nitrate is used, trial 5 to 9 mmol/day and confirm that antibacterial mouthwash is not used near dosing. | Reduce uncertainty and confirm that nitrate timing, taste, gastrointestinal tolerance, and mouthwash restrictions are practical. |
| 3 to 7 days before competition | Optional nitrate loading at 5 to 9 mmol/day. Optional multi-day bicarbonate protocol, generally 0.4 to 0.5 g/kg/day in divided doses, when acute bicarbonate causes gastrointestinal symptoms. | Elevate nitrate/nitrite availability and, if used, distribute bicarbonate exposure to reduce single-dose gastrointestinal burden. |
| Day before competition | Maintain normal carbohydrate availability and hydration. Avoid unusually high fiber, fat, alcohol, or unfamiliar foods. | Preserve glycogen and reduce gastrointestinal risk. |
| 2 to 3 hours before competition | If nitrate is used, take the final nitrate bolus so that peak nitrite availability is expected during competition. | Target nitrate-nitrite-NO physiology during the high-intensity effort. |
| 60 to 180 minutes before competition | If acute bicarbonate is used, take 0.2 to 0.3 g/kg with a carbohydrate-containing meal or in divided boluses. Timing should be based on prior individual testing. | Increase extracellular bicarbonate, blood pH, base excess, and H+ efflux capacity while minimizing gastrointestinal symptoms. |
| 30 to 60 minutes before competition | Use caffeine, commonly 2 to 3 mg/kg for conservative use or 3 to 6 mg/kg in athletes who tolerate it well. | Increase arousal, motor output, effort tolerance, and pain tolerance without excessive side effects. |
| Between heats, if applicable | Use fluids and carbohydrate as needed. During prolonged competition windows, small frequent carbohydrate doses may be preferable to one large bolus. | Maintain blood glucose, preserve substrate availability, and reduce late-session perceptual strain. |

Case Scenario 2 Repeated Sprint Sport

**Decision status:** This scenario is intended as a model-based example, not an individualized prescription. The full sequence should be tested in training or simulated competition before use in competition.

**Scenario:** A field or court sport athlete, such as a soccer winger, rugby sevens player, basketball guard, or lacrosse midfielder, must complete repeated accelerations, sprint bouts, changes of direction, and skill actions across a match or tournament with incomplete recovery.

**Dominant model pressure:** The task repeatedly perturbs PCr, Pi, pH, local oxygen delivery, afferent feedback, effort, and pain. Recovery intervals partially restore PCr and reduce phosphate-linked carryover. The best supplement strategy should support phosphagen availability and recovery, reduce unnecessary metabolic strain, maintain carbohydrate availability, and improve arousal and tolerance.

**THEORETICAL BEST-FIT SUPPLEMENT HIERARCHY**

- Creatine is the most direct phosphagen-focused strategy. It may improve PCr availability, repeated high-power output, and between-bout PCr/Pi recovery in intermittent maximal exercise [45-48].
- Carbohydrate is foundational when the match, practice, or tournament is long enough for glycogen, blood glucose, or late-session skill and effort responses to matter [63-66].
- Caffeine is useful when vigilance, sprint effort, decision-making, and tolerance are important, provided that sleep, anxiety, tremor, and gastrointestinal tolerance are managed [60-62].
- Dietary nitrate is a conditional repeated-sprint strategy, especially when the protocol has short recoveries and high type II fiber demand. It should not be assumed effective for all sprint durations or recovery structures [37,39,41].
- Sodium bicarbonate and beta-alanine are conditional additions when repeated bouts are sufficiently glycolytic, when repeated-sprint density is high, or when the athlete competes in tournament formats with repeated intense phases [50-59].

TABLE S3

Competition-timing plan for repeated sprint sport

| Timing | Suggested strategy | Mechanistic purpose |
| --- | --- | --- |
| 4 to 8 weeks before competition | Begin creatine. A rapid option is 20 g/day for 5 to 7 days in four divided doses, followed by 3 to 5 g/day. A slower option is 3 to 5 g/day for 3 to 4 weeks. If beta-alanine is used, begin 4 to 6 g/day. | Increase total creatine and PCr availability. Build intracellular buffering if repeated glycolytic work is a major part of the sport or training block. |
| 2 to 4 weeks before competition | Test the caffeine dose, carbohydrate plan, and any bicarbonate protocol in full-speed practice. Monitor sleep and gastrointestinal symptoms. | Ensure that the practical stack improves repeated output without increasing aversive sensations that could impair pacing, decision-making, or willingness to sprint. |
| 3 to 7 days before competition | Optional nitrate loading at 5 to 9 mmol/day. Optional multi-day bicarbonate protocol if the athlete benefits from alkalosis but poorly tolerates acute dosing. | Prepare nitrate-nitrite availability for short-recovery sprint work and, if used, support extracellular buffering with lower single-dose gastrointestinal burden. |
| 24 hours before competition | Prioritize adequate carbohydrate intake, fluids, sodium from normal foods, and sleep. Avoid first-time supplement use. | Maximize carbohydrate availability and reduce avoidable non-exercise fatigue. |
| 1 to 4 hours before competition | Consume a carbohydrate-rich pre-event meal, commonly 1 to 4 g/kg depending on meal timing, event length, and gut tolerance. | Support liver glycogen, muscle glycogen, blood glucose, and sustained match output. |
| 2 to 3 hours before competition | If nitrate is used, take the final nitrate bolus and avoid antibacterial mouthwash around dosing. | Support nitrate-nitrite-NO physiology during match play. |
| 60 to 180 minutes before competition | If bicarbonate is used acutely, use 0.2 to 0.3 g/kg, ideally with a carbohydrate-containing meal and only after successful practice trials. | Support extracellular buffering during repeated high-glycolytic bouts while reducing gastrointestinal risk. |
| 30 to 60 minutes before competition | Use caffeine at an individualized dose, often near 3 mg/kg. Higher doses should be reserved for athletes with demonstrated tolerance and a clear need. | Support arousal, sprint willingness, vigilance, and tolerance. |
| During competition or halftime | Use carbohydrate-containing fluids, gels, or foods as tolerated. For long matches or tournament blocks, 30 to 60 g/hour is a practical target range. | Maintain blood glucose, support repeated high-intensity work, and reduce late-session perceptual and skill decrements. |
| Post-match or between matches | Use carbohydrate plus fluid and electrolytes soon after exercise. Continue creatine maintenance if used. | Accelerate refueling and recovery for repeated competition exposures. |

Case Scenario 3 Combat Sport

**Decision status:** This scenario is intended as a model-based example, not an individualized prescription. The full sequence should be tested in training or simulated competition before use in competition.

**Scenario:** A judo, Brazilian jiu-jitsu, wrestling, boxing, or mixed martial arts athlete is preparing for repeated rounds or multiple tournament bouts. The event combines repeated glycolytic flurries, isometric gripping or clinching, high arousal, pain, tactical decision-making, and incomplete recovery between rounds or bouts.

**Dominant model pressure:** Combat sport creates overlapping acid-base stress, Pi-linked fatigue, pain, arousal, and recovery constraints. The most plausible strategy targets intracellular buffering, extracellular buffering and H^+^ efflux, central arousal and pain tolerance, and carbohydrate availability across repeated bouts.

**THEORETICAL BEST-FIT SUPPLEMENT HIERARCHY**

- Beta-alanine plus sodium bicarbonate is the strongest mechanistic pairing when repeated glycolytic efforts and upper-body fatigue dominate. Beta-alanine targets intracellular buffering, whereas bicarbonate targets extracellular buffering and H^+^ efflux [50,54,58,59].
- Caffeine is useful when arousal, aggression control, vigilance, motor output, and pain tolerance need to be optimized. The dose should be conservative in athletes prone to anxiety, tremor, or sleep disruption [60-62].
- Carbohydrate is important for tournaments, long training sessions, repeated weigh-in recovery windows, and bouts separated by short recovery periods [63-66].
- Creatine is plausible for repeated high-force efforts and training adaptation, but it should be used only when body mass, weigh-in rules, and water shifts are compatible with the athlete's weight-class plan [45-48].
- Dietary nitrate is an optional secondary strategy. It may be worth testing when repeated flurries and incomplete recovery create a pattern similar to intermittent severe-intensity work, but evidence is less direct than for buffering, caffeine, carbohydrate, and creatine in this phenotype [37,39,41].

TABLE S4

Competition-timing plan for combat sport

| Timing | Suggested strategy | Mechanistic purpose |
| --- | --- | --- |
| 6 to 10 weeks before competition | Begin beta-alanine at 4 to 6 g/day. If creatine is compatible with the weight-class plan, begin loading or daily maintenance early enough to monitor body mass. | Increase muscle carnosine and determine whether creatine-associated mass changes are acceptable before weight management becomes restrictive. |
| 3 to 6 weeks before competition | Practice the complete supplement stack during hard sparring or simulated tournament rounds. Test caffeine dose and bicarbonate timing. Record gastrointestinal symptoms, perceived effort, pain, and round-to-round output. | Confirm that theoretical benefits translate to the athlete's actual combat-sport demands. |
| 7 days before competition | If bicarbonate is used, consider a multi-day divided-dose protocol or an acute individualized protocol. Avoid new products. If nitrate is used, begin 3 to 7 days of nitrate loading. | Support extracellular buffering while reducing single-dose side effects. Elevate nitrate availability if the athlete has previously responded. |
| Before weigh-in | Avoid supplements that threaten the weight target, gastrointestinal comfort, or hydration status. Creatine and bicarbonate sodium load should be managed as part of the overall weight-class plan. | Prevent theoretically useful supplements from compromising weigh-in success or competition readiness. |
| After weigh-in, when applicable | Prioritize rehydration, carbohydrate, electrolytes, and familiar low-residue foods. Bicarbonate should be used only if the athlete has enough time and prior evidence of tolerance. | Restore fluid and carbohydrate availability without adding gastrointestinal distress. |
| 1 to 4 hours before first bout | Consume a carbohydrate-containing meal or snack adjusted to gut tolerance and bout timing. For tournament days, plan repeated small carbohydrate feedings rather than one large meal. | Support repeated high-intensity output and central/perceptual resilience across bouts. |
| 2 to 3 hours before first bout | If nitrate is used, take the final dose and avoid antibacterial mouthwash around dosing. | Target nitrate-nitrite-NO physiology during intermittent high-intensity work. |
| 60 to 180 minutes before first bout | If acute bicarbonate is used, take 0.2 to 0.3 g/kg at the individualized time-to-peak. Split dosing and carbohydrate co-ingestion may improve tolerance. | Increase extracellular buffering and H^+^ efflux capacity during repeated flurries. |
| 30 to 60 minutes before first bout | Use caffeine conservatively, often 1 to 3 mg/kg for athletes prone to anxiety or 3 to 6 mg/kg only when tolerance is well established. | Support arousal, motor output, and pain tolerance while avoiding tremor or excessive sympathetic activation. |
| Between bouts | Use fluids plus small carbohydrate doses as tolerated. Continue to avoid unfamiliar products. If caffeine top-ups are used, they should be small and previously tested. | Preserve blood glucose, maintain alertness, and support repeated bout performance without impairing sleep or gastrointestinal comfort. |

IMPLEMENTATION NOTES

The supplement strategy should be matched to the limiting phenotype, not to the sport name alone. The same sport can include athletes with different fatigue pressures depending on role, event duration, recovery windows, training status, and pacing strategy.

No supplement should be used for the first time in competition. The full stack should be tested in the same environmental, meal-timing, and performance context expected on competition day.

Bicarbonate and nitrate timing can overlap. When both are used, athletes should practice the full timing sequence, such as nitrate approximately 2 to 3 hours before competition and bicarbonate at the athlete's individualized 60 to 180 minute pre-event window.

Caffeine should not be maximized by default. Lower doses may be preferable when anxiety, tremor, tactical control, or sleep are important.

In weight-class sport, theoretical ergogenic benefit is secondary to safe weight management, rehydration, gastrointestinal comfort, and weigh-in compliance.

These examples should be presented as theoretical applications of the model. They are useful for hypothesis generation and applied interpretation, but they should not be interpreted as proof that supplement combinations are uniformly additive or that maximal stacking is warranted.
